# How to prioritize treatment in dual malignancy: A case report of patient with non-Hodgkin lymphoma and breast cancer

**DOI:** 10.1016/j.amsu.2022.104300

**Published:** 2022-07-31

**Authors:** Alvi Chomariyati, Muhammad Noor Diansyah, Putu Niken Ayu Amrita, Pradana Zaky Romadhon, Siprianus Ugroseno Yudho Bintoro, Ami Ashariati, Merlyna Savitri

**Affiliations:** a Department of Internal Medicine, Faculty of Medicine, Airlangga University, Surabaya, Indonesia; b Staff Member of Hematology and Medical Oncology Division, Department of Internal Medicine, Dr. Soetomo General Teaching Hospital, Surabaya, Indonesia; c Head of Hematology and Medical Oncology Division, Dr. Soetomo General Teaching Hospital, Surabaya, Indonesia

**Keywords:** NHL, Breast cancer, Multiple, Primary malignancies, Medicine

## Abstract

**Introduction:**

Clinicians often encounter dilemma upon treating multiple primary malignancies.

**Case presentation:**

We report a case of a female patient, 72, complained of a lump under her left eye since January 2019. Patient was diagnosed with infiltrating ductal carcinoma grade III of the right breast in June 2018 with ER+, PR+, and HER2-, treated with hormonal treatment. Histopathology examination of the lump revealed Non Hodgkin Lymphoma (NHL), B cell type, high grade. Patients received rituximab, cyclophosphamide, epirubicin, vincristin, and prednisone (RHCOP) for 6 cycles to overcome lymphoma then received hormonal therapy afterwards.

**Clinical discussion:**

According to earlier published case reports, it's advised to start hormonal therapy after RHCOP. The survival time was 21 months (5.1–114.7 months) with 5-year overall survival 29%

**Conclusion:**

Unfortunately, we could not have a follow-up on the patient after finishing 6 cycles of RHCOP due to the COVID-19 pandemic situation.

## Highlights

• A patient may experience multiple primary malignancies.
• Non-hodgkin lymphoma (NHL) as the secondary malignancy in BC is a rare case.
• In a BC patient with secondary NHL, we should consider to treat NHL first using anthracycline-based treatment and rituximab, in this case, RHCOP, if the CD20 turns out positive.
• Hormonal therapy would be the next agenda.

## 1 Introduction

In the population, multiple primary malignancies have increased in number over the past decade. The prevalence of multiple primary malignancies worldwide is 0.734–11.7%. There are 14–20% of patients with primary malignancy with higher risk to experience multiple primary malignancies affecting their quality of life [[1], [2], [3]]. Male and elderly patients majorly undergo this situation [4]. The ratio of metachronous to synchronous is 2.7:1, with breast (15.6%) and lung (22.9%) as the main localizations. It's seldom to find non hodgkin lymphoma (NHL) accompanying right breast cancer (BC). To date, there is still no established guideline [2,5].

Referring to SCARE 2020 guidelines [6], below we present a case about the management of a patient with non-hodgkin lymphoma (NHL) and right breast cancer (BC).

## 2 Case illustration

Mrs. M, aged 72, Javanese, married, a housewife, lives in Banyuwangi, East Java presented to the Eye Clinic at a public hospital on April 1st^,^ 2019 with a painless lump under her left eye since January 2019. No visual disturbances complained. No lumps found on other parts of the body. In early 2018, she had a lump in right breast which was then surgically removed in June 2018. The patient never had chemotherapy before. The infraorbital lump occurred 6 months following the breast lump. She now has menopause.

**Fig. 1:**
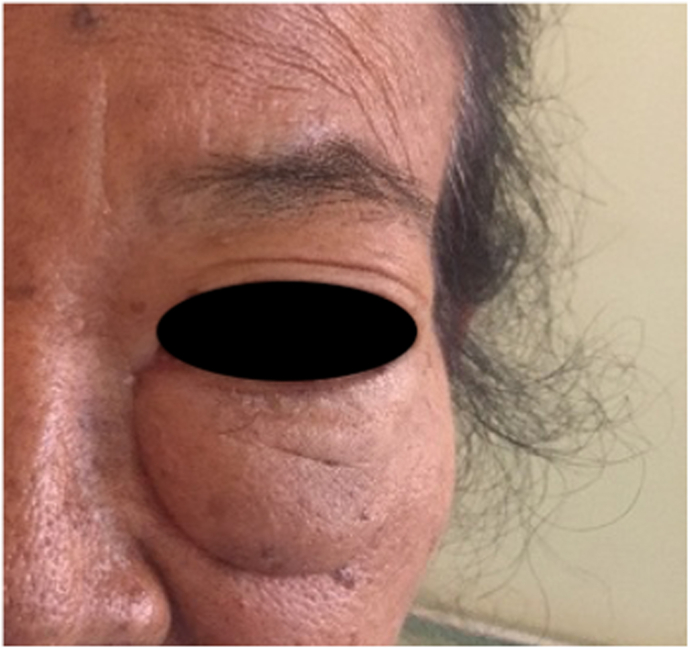
Lump in the left eye.

A mass, 5 × 3 cm in size, located under the left eye (infraorbital). The mass was skin-colored, soft, mobile, and painless (Fig. 1). Visual examination showed no impairment. There was no increase in jugular venous pressure and neck lymph node enlargement. There was mastectomy scar on right breast.

**Fig. 2:**
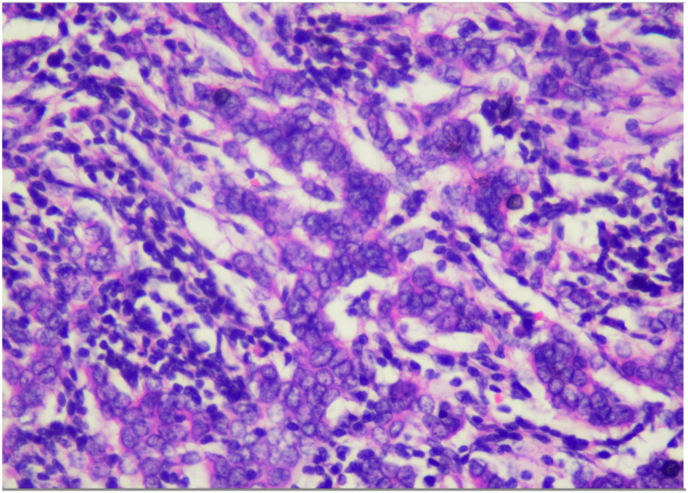
Histopathology analysis of right breast lump: invasive carcinoma of no special type grade III, papilla and skin epidermis were tumor-free region, no lymph nodes invasion and metastasis.

Histopathology examination of the lump on the right breast revealed infiltrating ductal carcinoma, grade III; no axillary lymph node metastases; no blood vessel and skin invasion, and malignant cell-free surgical base (See Fig. 2.). Immunohistochemistry (IHC) examination revealed ER positive, moderate to strong intensity in 80%–90% of tumor cells; PR positive, moderate to strong intensity, heterogeneous feature in 40%–50% of tumor cells; HER2 was weakly expressed with only atypical membranous pattern, barely perceivable, categorized as 1+ score considered as negative, and Ki67 index proliferation is 70%.

**Fig. 3:**
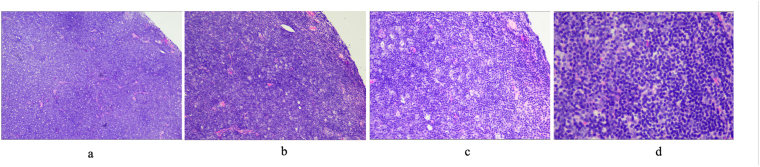
Histopathology examination of lump tissue under the left eye under various magnifications: **a) 40× b) 100x c) 200x d) 400x**.

**Fig. 4:**
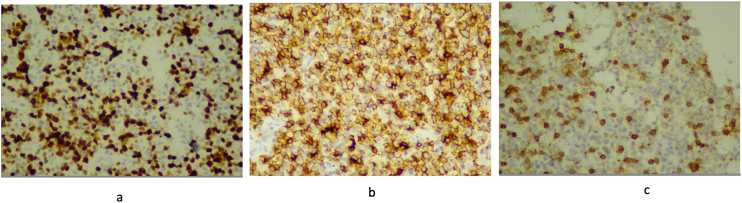
Immunohistochemistry evaluation on tissue from the left eye lump **a) CD3**: membrane cell: negative; lymphocyte T: positive; **b) CD20**: membrane cell: positive; **c) Ki67**: proliferative index 70%.

Histopathological examination of the left eye done at at a public hospital obtained pieces of tumor tissue arranged in a diffuse pattern, consisting of a proliferation of round-nucleated anaplastic lymphoid cells, relatively monotonous, hyperchromatic, mitotic 20/10 HPF with IHC results CD3 negative on tumor cell membranes; CD20: positive on tumor cell membranes; Ki67: proliferation index 70%, with the conclusion: Non Hodgkin Lymphoma, B cell type, high grade. (See Fig. 3, Fig. 4).

Patient was diagnosed with right infiltrating ductal carcinoma (luminal A) BC grade III stage IIA T2N0M0. Patient underwent radical mastectomy (MRM) with positive ER positive and PR detected, but negative for HER2, and left infraorbital NHL, B cell type high grade, CD20^+^, stage IAE. After multidisciplinary discussion, patient received rituximab, cyclophosphamide, epirubicin, vincristine, and prednisone (RHCOP) (day 1–5), followed with hormonal therapy after 6 cycles of RCHOP were completed. However she was not able to continue the follow-up program due to the unfavourable pandemic COVID-19 situation. Besides, she reached the state when she barely complained symptoms regarding both breast and eye lump. The patient showed a satisfactory response after completing the second cycle of chemotherapy then proceeded with the seventh cycle. Both early and late chemotherapy complications were not documented. Follow-up was done without further oncological treatment. The patient maintains a good performance and functional status.

## 3 Discussion

Clinically, multiple primary malignancies often confuse clinicians since both recurrence and distant metastasis of the primary tumor since are characterized by new lesions. Metastatic tumors originate from the primary lesion with both exhibiting similar pathological characteristics and similar developmental processes and prognoses, whereas multiple primary malignancies refer to the development of a new malignant tumor, with completely distinct properties [7]. Furthermore, secondary malignancy is a malignant tumor induced by the preceding treatment (usually radiation or chemotherapy). However, BC patients have a higher risk for developing a second malignancy after adjuvant chemotherapy and radiotherapy, including leukemia and non-Hodgkin's lymphoma [8]. Based on Warren and Gates criteria, multiple primary malignancies should have clear evidence of malignancy on histologic examination in which each tumor should be separated from one organ, and the metastatic lesions should be excluded [2,4]. In this case, we figure out pathologically-confirmed NHL as the secondary malignancy.

Currently, there are no guidelines available for the treatment of (NHL) as the secondary malignancy. The type of malignancy, stage of disease, and the patient's general condition are points to consider when determining multidisciplinary tailored treatment [4]. Ultimately, patients should always also be informed about the therapeutic challenges and prognosis. Several therapeutic approaches are being adapted to figure out new strategies without increasing toxicity [3].

A study in Korea on more than 80.000 BC patients within 2002–2016 revealed that1564 of them experienced NHL, including diffuse large B-cell lymphoma (DLBCL), follicular lymphoma (FL), mature T/NK-cell lymphomas, anaplastic large cell lymphoma (ALCL), and unspecified types of NHL. The fully adjusted hazard ratio (HR) for NHL associated with the development of BC was 1.64 (95% CI = 1.34–2.00) after adjusting to body mass index, alcohol intake, physical activity, smoking, income, and comorbidity. The adjusted HR for NHL was much higher in participants aged <50 years and who received hormonal therapy (either tamoxifen or aromatase inhibitors) than in them ≥50 years or who did not receive hormone therapy, respectively [9].

Furthermore, a case report presenting BC patient with secondary NHL disclosed the treatment plan consisting of anthracycline-based treatment and rituximab if CD20 turns out positive [10]. (See Table 1). The other case report showed that NHL as secondary breat cancer can show complete remission but relapse in two years. Based on Vignot et al., the survival time was 21.5 months (5.1–114.7 months) with 5-year overall survival was 29% [11]. The response rates and the survival times may seem disappointing due to the initial treatment, such as tumorectomy or mastectomy [12].

**Table 1:** Reports of non-hogkin lymphoma (NHL) as the secondary malignancy of breast cancer and treatment strategies.

Author	Year	Country	Treatment strategy	Result
Zhong et al. [9]	2014	China	anthracycline-based treatment and rituximab if CD20 positive (R–CHOP)	Not mentioned
Nagasaki et al. [11]	2003	Japan	EPOCH G	Complete remission of NHL and relapse after 2 years

R–HCOP, Rituximab-cyclophosphamide, doxorubicine, vincristine, prednisone; EPOCH, etoposide-prednisone-Oncovin-cyclophosphamide-hydroxydaunorubicin regimen.

In this case, deciding the treatment is challenging and often a dilemma. We agreed to administer RHCOP first, then proceed with the hormonal therapy, based on the established literature. It is regrettable that we could not monitor the progress since COVID-19 pandemic attacked.

## 4 Conclusion

NHL as the secondary malignancy in BC is a rare case. In a BC patient with secondary NHL, we should consider to treat the NHL first using anthracycline-based treatment and rituximab, which is RHCOP, if the CD20 turns out positive. Hormonal therapy would be the next treatment plan. Unfortunately, monitoring the treatment outcome in this case was not possible due to the COVID-19 pandemic.

## Ethical approval

The patient in this study has stated the approval.

## Sources of funding

No funding sponsor involved in this study.

## Author contribution

All the authors have accepted responsibility for the entire content of this submitted manuscript and approved submission. AC, MS provided the study concept and design, interpreted the study data, and critically reviewed the intellectual con-tent of the manuscript. AA and SUY led and supervised the study conduct. AC actively involved in the took care of the study subjects and acquired the study data. All authors made the decision to submit the manuscript for publication. All authors have access to the data and assume responsibility for the integrity and completeness of the reported data and to maintain confidentiality of the data.

## Trail registry number

1. Name of the registry:

2. Unique Identifying number or registration ID:

3. Hyperlink to your specific registration (must be publicly accessible and will be checked):

## Guarantor

Merlyna Savitri.

Jl. Mayjen Prof. Dr. Moestopo No.47, Pacar Kembang, Kec. Tambaksari, Kota SBY, Jawa Timur 60132

merlyna.savitri@gmail.com

Accepts full responsibility for the work and/or the conduct of the study, had access to the data, and controlled the decision to publish.

## Annals of medicine and surgery

The following information is required for submission. Please note that failure to respond to these questions/statements will mean your submission will be returned. If you have nothing to declare in any of these categories then this should be stated.

## Consent

Studies on patients or volunteers require ethics committee approval and fully informed written consent which should be documented in the paper.

Authors must obtain written and signed consent to publish a case report from the patient (or, where applicable, the patient's guardian or next of kin) prior to submission. We ask Authors to confirm as part of the submission process that such consent has been obtained, and the manuscript must include a statement to this effect in a consent section at the end of the manuscript, as follows: "Written informed consent was obtained from the patient for publication of this case report and accompanying images. A copy of the written consent is available for review by the Editor-in-Chief of this journal on request”.

Patients have a right to privacy. Patients’ and volunteers' names, initials, or hospital numbers should not be used. Images of patients or volunteers should not be used unless the information is essential for scientific purposes and explicit permission has been given as part of the consent. If such consent is made subject to any conditions, the Editor in Chief must be made aware of all such conditions.

Even where consent has been given, identifying details should be omitted if they are not essential. If identifying characteristics are altered to protect anonymity, such as in genetic pedigrees, authors should provide assurance that alterations do not distort scientific meaning and editors should so note.

Written informed consent was obtained from the patient for publication of this case report and accompanying images. A copy of the written consent is available for review by the Editor-in-Chief of this journal on request.

## Provenance and peer review

Not commissioned, externally peer-reviewed.

## Declaration of competing interest

Authors declare no conflict of interest.
